# Persistent Need for Ophthalmic Follow-Up After Simultaneous Pancreas–Kidney Transplantation: Long-Term Effects on Diabetic Retinopathy and Quality of Life

**DOI:** 10.3390/jcm14217779

**Published:** 2025-11-02

**Authors:** Joonas Wirkkala, Risto Ikäheimo, Anna-Maria Kubin, Pasi Ohtonen, Nina Hautala

**Affiliations:** 1 Research Unit of Clinical Medicine, University of Oulu, 90220 Oulu, Finland; joonas.wirkkala@oulu.fi (J.W.); anna-maria.kubin@oulu.fi (A.-M.K.); 2 Department of Ophthalmology, Oulu University Hospital, 90220 Oulu, Finland; 3 Medical Research Center, Oulu University Hospital, 90220 Oulu, Finland; 4 Department of Nephrology and Internal Medicine, Oulu University Hospital, 90220 Oulu, Finland; risto.ikaheimo@fimnet.fi; 5 Research Service Unit, Oulu University Hospital, 90220 Oulu, Finland; pasi.ohtonen@oulu.fi; 6 Research Unit of Surgery, Anesthesiology and Intensive Care, University of Oulu, 90220 Oulu, Finland

**Keywords:** simultaneous pancreas–kidney transplantation, diabetes, diabetic retinopathy, diabetic nephropathy

## Abstract

**Background**: Simultaneous pancreas–kidney transplantation (SPKT) is an effective treatment for patients with type 1 diabetes (T1D) and end-stage kidney disease (ESKD). However, its long-term impact on diabetic retinopathy (DR) stability is not fully understood. This study evaluated DR severity, visual outcomes, and health-related quality of life (HRQoL) in patients with T1D post-SPKT. **Methods**: This quantitative longitudional study included 24 patients with T1D and ESKD who underwent SPKT between 2013 and 2020. Data included HbA1cand creatinine levels, comprehensive ophthalmic evaluations with fundus imaging, and HRQoL assessment using the 15D instrument. **Results**: Eighteen patients completed follow-up. The mean age at SPKT was 39 ± 7 years, with 67% male. Post-SPKT, HbA1c and creatinine improved significantly among all participants. The mean ETDRS letter gain was 5.2 letters (95% CI 0.03 to 10.29; *p* = 0.049). Cataract progression occurred in 39% of phakic eyes (*p* < 0.001), and seven patients had previous cataract surgery. Seventeen (89%) patients had proliferative DR (PDR) pre-SPKT, with 28% progressing to sight-threatening DR post-SPKT (*p* = 0.037). One patient (6%) was visually impaired. HRQoL scores were comparable to controls, though patients with PDR had lower vision-related scores (*p* = 0.023). **Conclusions**: Despite metabolic improvements after SPKT, 28% of patients experienced DR progression to severe PDR, highlighting the need for long-term ophthalmic follow-up.

## 1. Introduction

The global diabetes epidemic is affecting an increasing number of individuals across the world. The International Diabetes Federation project has estimated that the population with diabetes will rise to 783 million by the year 2045 [1]. Diabetic retinopathy (DR) is the most common microvascular end-organ complication of diabetes, occurring in 30% to 40% of all individuals with diabetes, and the rise in diabetes prevalence is clearly paralleled in DR [2]. DR is a leading cause of irreversible visual loss, which can be prevented through early detection by effective fundus-photography-based screening programs and timely intervention of sight-threatening DR with laser treatment, intravitreal anti-VEGF therapy or surgical interventions [3]. Diabetes duration and glycemic control are associated with the development and progression of DR, although the multifactorial pathogenesis of DR is not fully understood. It is well accepted that management of systemic disease with strict regulation and treatment of hyperglycemia, hypertension, and hyperlipidemia is the first step in delaying onset and progression of DR.

As diabetes continues to be a worldwide epidemic, diabetic chronic kidney disease (CKD) remains the single most important cause of end-stage kidney disease (ESKD). CKD in individuals with diabetes is diagnosed by the persistent elevation of urinary albumin excretion (albuminuria), low estimated glomerular filtration rate (eGFR), or other manifestations of kidney damage in the absence of signs or symptoms of other primary causes of kidney damage. Approximately one-third of individuals with type 1 diabetes (T1D) develop CKD, and the prevalence of ESKD in T1D increases with long-standing duration of diabetes, similarly to the risk of DR [4]. Substantial increases in morbidity and mortality along with marked rise in treatment costs and marked reduction in quality of life (QoL) are the usual consequences of onset of CKD and progression to ESKD in patients with T1D. Simultaneous pancreas–kidney transplantation (SPKT) is a promising treatment option for patients with both T1D and advanced ESKD. By restoring euglycemia and improving kidney function, SPKT has the potential to influence diabetic-related complications, including DR. However, despite the proven metabolic benefits of SPKT, there is a risk of DR progression in some patients.

Previous studies have shown some controversial results of the impact of SPKT on DR [5,6,7,8,9]. While SPKT has been shown to stabilize and even improve the course of DR in many patients [7,10,11,12], including reduced active vascular proliferation, decreased need for retinal laser photocoagulation, and vitrectomy procedures [13,14], most studies have reported the SPKT outcomes on DR after a relatively short follow-up periods. Consequently, the long-term influence of SPKT on DR stability is not fully understood.

Both T1D and DR have been shown to exert emotional and social impacts, potentially altering individuals’ perceptions of their health-related quality of life (HRQoL) [15,16]. Severe hypoglycemia remains a persistent challenge for individuals with T1D throughout their lifespan, while DR progression further impairs daily functioning and has been associated with poor glycemic control [17]. There is substantial evidence that maintaining excellent glycemic control over three decades significantly reduces the incidence of diabetes-related complications and mortality, while also improving HRQoL [18]. Although there has been considerable progress in the development of insulin delivery devices, newer insulin molecules, and glucose monitoring systems in recent years, there is still a significant negative impact on the QoL in relation to disease management among patients with T1D. SPKT offers a means to restore euglycemia in patients with diabetes. However, the high risk of surgical complications and the lifelong requirement for immunosuppressive therapy may influence HRQoL post-transplant, despite evidence of overall HRQoL improvement following SPKT [19,20]. Lack of wide availability, high cost, infections, and immunological problems following SPKT have long been the challenges precluding its widespread use. However, advanced surgical techniques and newer immunosuppressive drug regimens in recent years have resulted in dramatic improvement in treatment outcomes of SPKT.

The aim of this prospective-retrospective longitudional study was to evaluate the long-term effects of SPKT on DR and its progression, visual acuity, ocular comorbidities, blood glucose and serum creatinine levels, and HRQoL in patients with T1D.

## 2. Materials and Methods

All SPKT procedures in Finland are performed at Helsinki University Hospital [21], however, patient selection and follow-up are carried out at local hospitals. This consecutive case series included all patients who underwent SPKT by December 2020 within the Northern Ostrobothnia Healthcare District. As previously described, pre-operative workup consisted of cardio-pulmonary evaluation with spirometry, echocardiogram, and coronary angiography or myocardial perfusion scan. Electroneuromyography, computed tomography of abdomen, and ultrasound of carotid arteries were performed to assess possible diabetic neuropathy and evaluate status of abdominal/carotid calcifications. In addition, comprehensive pre-transplant laboratory work-up included coagulation status (thrombin time, activated partial thrombin time, antithrombin 3, fibrinogen, D-dimer, and factor VIII) [21]. All patients had ophthalmic control visits within 6 months before transplantation, at 6 months after surgery, and then annually. Ophthalmologist evaluated the severity of DR prior to entry and in cases of active proliferative diabetic retinopathy (PDR) panretinal photocoagulation (PRP) was performed. The participants of the current study were followed for at least two years and participated in the study visit at Oulu University Hospital. The study was conducted according to the declaration of Helsinki and was given approval by the Ethical Research Committee of Oulu University Hospital in 2018. Informed consent was obtained from all participants. Some patients had previously undergone SPKT as early as 2013, and data pertaining to their surgical procedures were retrospectively extracted from medical records after the study approval. Nevertheless, all patients included in the study underwent prospective ophthalmologic assessments at Oulu University Hospital between 2018 and 2020, subsequent to the study’s approval. A comprehensive ophthalmologic evaluation included measurements of best-corrected visual acuity (BCVA) and intraocular pressure (IOP), optical coherence tomography (OCT), fundus photography and slit-lamp examination in mydriasis, performed both pre-SPKT and at the study visit. The severity scale of DR was defined as non-DR, mild nonproliferative diabetic retinopathy (NPDR; presence of microaneurysms; no other signs of retinopathy), moderate NPDR (more severe retinal changes, including cotton wool spots and intraretinal hemorrhages, but no signs of neovascularization), severe NPDR (at least one of the following is present: hemorrhages in four quadrants, venous beading in two or more quadrants, intraretinal microvascular abnormalities in one or more quadrants), and PDR (defined by the presence of neovascularization on the optic disc or elsewhere in the retina, which can lead to serious complications such as vitreous hemorrhage or retinal detachment). Glycemic control (HbA1c) and creatinine were measured before SPKT, one month after and every 6 months thereafter. Low-density lipoprotein (LDL) cholesterol was measured before SPKT and then at the study visit. HRQoL was evaluated using the 15D instrument [22], and scores were compared with healthy age- and sex- matched controls. The 15D is a generic, comprehensive, 15-dimensional, standardized, self-administered measure of HRQoL that can be used both as a profile and single index score measure. It is a tool designed to assess health-related quality of life across 15 different dimensions. These dimensions include physical health, mental well-being, social functioning, emotional well-being, and other aspects of daily life and health. Participants respond to questions about their health in various domains, using a scale from 0 to 1. The single index (15D score) on a 0–1 scale, representing the overall HRQoL (0 = being dead, 0.0162 = being unconscious or comatose, 1 = no problems on any dimension = ‘full’ HRQoL) is calculated from the health state descriptive system by using a set of population-based preference or utility weights. Paired *t*-tests were used for continuous pre- and post-SPKT comparisons, and χ^2^-tests for categorical outcomes. Longitudinal changes in HbA1c and serum creatinine were assessed with linear mixed-effects models. A two-sided *p* < 0.05 was considered statistically significant. Ophthalmic outcomes were analyzed from both eyes of a single patient, when interocular differences were presented, data from the worse eye was used. The criteria for significant DR progression were defined as a change in more than one class in DR severity scales or a change from stable DR to sight-threatening DR.

## 3. Results

Of the 24 patients who underwent SPKT, 18 (75%) completed the follow-up examinations, while six (25%) declined to participate. No patients were excluded due to graft failure or death. A total of 24 patients with T1D underwent SPKT, of whom 18 (75%) completed follow-up examinations. The follow-up duration was at least 2 years, with a mean follow-up time of 4.6 ± 2.6 years and a maximum of 10 years. The mean age at SPKT was 39 ± 7 years, and 12 (67%) patients were male. The mean duration of preceding dialysis was 1.5 ± 1.2 years. Clinical characteristics of the participants are presented in Table 1.

All patients were diagnosed with DR pre-SPKT, with 16 (89%) having PDR. Following SPKT, significant DR progression was observed in 7 patients. Among them, 5 patients (28%) progressed from mild NPDR or inactive PDR to active PDR (χ^2^-test, comparing pre- vs. post-SPKT DR severity, *p* = 0.037). An additional 2 patients experienced less severe DR progression but still required treatment for DR. Treatments included macular laser in one patient (6%), panretinal photocoagulation in 2 (11%), and intravitreal anti-VEGF injection alone or in combination with laser in 4 patients (22%). None of the study participants developed vitreous hemorrhages or required pars plana vitrectomy. The mean time from SPKT to DR progression was 18 months, ranging from 3 months to almost 6 years. DR was stable or improved without further ophthalmic treatment in 11 out of 18 patients (61%) (Table 2).

At the study visit, mean BCVA in the worse eye was 69 ± 24 ETDRS letters, with only one patient (6%) classified as visually impaired according to WHO criteria (BCVA < 0.3 or ETDRS less than 59 letters in a better eye). Mean ETDRS letter gain post-SPKT was 5.2 letters (95% CI 0.03 to 10.29; paired t-test, *p* = 0.049). No significant change in central retinal thickness (CRT) was observed post-SPKT (mean difference 11.5 µm; 95% CI −4.7 to 27.7; paired t-test, *p* = 0.137). Prior to SPKT, 7 (39%) patients had undergone cataract surgery with intraocular lens (IOL) implantation, and a significant cataract progression was noted in 7 (39%) phakic eyes post-SPKT (χ^2^-test, *p* < 0.001). Ophthalmic outcomes are presented in Table 2.

**Figure 1:**
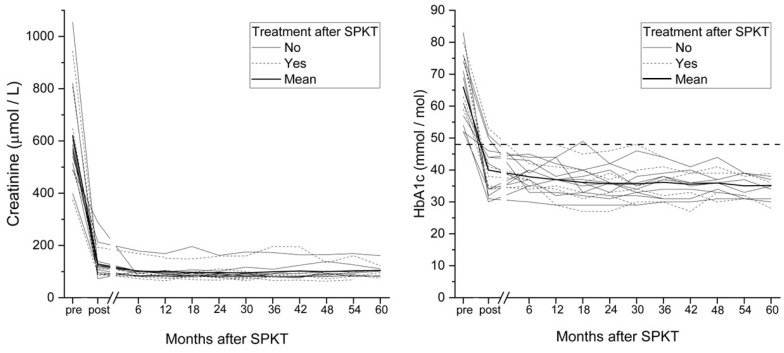
HbA1c and serum creatinine levels in patients with or without ophthalmic treatment following simultaneous pancreas–kidney transplantation.

All patients had successful SPKT, with significant and sustained improvements in HbA1c and creatinine, including patients who experienced DR progression (Figure 1). Standard immunosuppressive therapy included mycophenolate mofetil (MMF) and tacrolimus for all patients (Table 1). One patient (6%) experienced gastrointestinal adverse effects, necessitating a switch to azathioprine as the antiproliferative agent. Additionally, 3 patients (16%) required adjuvant prednisolone therapy. Graft dysfunction was observed in 27.8% of patients. However, the frequency of graft dysfunction did not differ between patients who required post-SPKT treatment for progression of DR and those who did not (*χ^2^*-test, *p* = 0.952).

**Figure 2:**
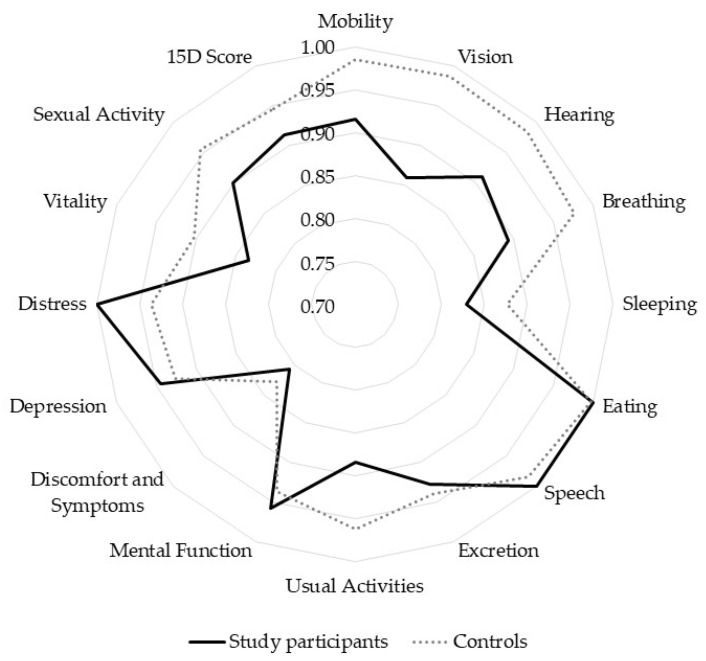
15D health-related quality of life measures in patients with type 1 diabetes after simultaneous pancreas–kidney transplantation and age- and sex-matched controls.

HRQoL, assessed by the 15D instrument (Figure 2), was comparable to healthy age- and sex-matched controls, with a total 15D score of 0.914 versus 0.946 (mean difference —0.033, 95% CI −0.072 to 0.007, *p* = 0.1, paired *t*-test). However, patients with PDR had a significantly lower vision-related dimension score compared to controls (0.86 vs. 0.99, mean difference —0.13, *p* = 0.023, paired *t*-test). The study participants received slightly higher scores for the 15D-dimensions for mental function and distress compared to age-matched, healthy controls.

## 4. Discussion

Significant improvement in BCVA and stability of DR was observed in most patients after SPKT during a long follow-up period of 2 to 10 years. On average, a significant 5.2-letter improvement in BCVA was achieved, regardless of DR stability. Two patients were visually impaired before SPKT, but only one met the criteria for visual impairment after transplantation. The improvement in vision is likely associated with the positive effects of stabilization of retinal glucose balance metabolism. Cataract progression was observed in 39% of phakic eyes, consistent with previous studies [7]. This finding is not unexpected, as the use of post-transplant immunosuppressants is known to increase the risk for cataract development.

Another key finding was the DR progression post-SPKT. While normalization of dysglycemia following SPKT generally slows microvascular complications, including DR, the extent of improvement may depend on DR severity at the time of transplantation. This may explain some differences between our results and previous studies [7,19]. Most of our study participants, 89%, had undergone panretinal photocoagulation for PDR, whereas in a previously published study, only half had any DR pre-SPKT [19]. Despite stable graft function and metabolic improvement, 28% of patients experienced DR progression from mild DR or inactive PDR to active PDR. In addition, irreversible capillary damage associated with PDR or inflammatory mechanisms that may persist independently of glucose levels could have contributed to the progression of DR among the study participants. Unlike some earlier studies, none of these study patients with progression to active PDR required vitrectomy [8,12]. Pre-transplant condition of the retina, particularly the level of metabolic control, likely influences post-transplant outcomes, and inadequate pre-transplant control may predispose to worse retinal outcomes even after achieving euglycemia.

The median time to DR progression post-SPKT was 9 months but ranged from 3 months to almost 6 years. One previous study revealed progression in more than one-third of patients within the first postoperative year [8]. In contrast, another study observed 5% DR progression during the same period [7]. These discrepancies may reflect differences in pre-transplant DR stability and metabolic control. In our study, metabolic control and kidney function improved and sustained throughout the follow-up period, confirming the long-term efficacy of SPKT in managing T1D. However, our results highlight that DR progression can still occur years after transplantation, underscoring the need for continuous ophthalmic follow-up even with stable graft function and metabolic control.

SPKT offers considerable benefits in terms of metabolic control and visual outcomes for patients with T1D and DR, as well as a positive impact on HRQoL [19,20]. Our results demonstrate that HRQoL scores of patients with T1D post-SPKT were comparable to healthy controls, although patients with PDR had lower vision-related scores, likely reflecting ongoing concerns requiring active monitoring and management. Improved vision contributes to maintaining proper functionality and HRQoL, regardless of the stability of DR after SPKT, as a decline in visual acuity has been shown to significantly impair HRQoL [23]. Interestingly, among the study patients, the 15D questionnaire results concerning mental well-being and distress were higher than those of the control group. This may be explained by satisfaction with the relief in everyday life after the procedure, without the need for constant insulin injections, blood glucose monitoring, and fear of the consequences of hypoglycemia.

Hyperglycemia plays a key role in DR development leading to damage of retinal blood vessels, but much remains to be further explored about the multifactorial pathogenesis of DR. Several mechanisms related to diabetes are known to involve in the pathogenesis of DR, primarily characterized by microvascular damage to the retina and resulting in vision-threatening damage if untreated. Other pathophysiological mechanisms in the development and progression of DR include oxidative stress, inflammation, and changes in retinal blood flow. Pericyte dysfunction may lead to capillary dropouts and subsequent retinal ischemia. Understanding the molecular mechanisms involved is crucial for developing new therapeutic strategies for DR. There is strong evidence that the course and severity of DR are related to the long-term metabolic control in patients with T1D, and improvements in metabolic control are crucial in prevention of complications [24]. The current therapeutic modalities of diabetes, including continuous glucose monitoring and advanced insulin delivery systems, enable tighter diabetes management [25,26]. However, recent study has shown that only one-fifth of the patients with T1D achieve the recommended HbA1c target of 53 mmol/mol [27]. Pancreas and islet transplantation remain the only approaches capable of reliably achieving near-physiological and robust glucose control, while SPKT specifically benefits patients with ESKD [28,29]. Despite the considerable specific risks, transplantation represents a lifesaving and life-enhancing option for carefully selected patients, although limited availability of the organs for transplantation, the need for long-term immunosuppression to prevent rejection, peri- and post-operative complications of SPKT, lack of resources and the expertise for the procedure in many centers, and the cost implications related to the surgery and postoperative care of these patients are major issues faced by clinicians worldwide.

Several limitations should be acknowledged, including the small sample size and single-center design, as well as variability in pre-SPKT DR treatment history, which could have influenced outcomes. Nevertheless, this study provides valuable long-term data for up to 10 years on visual outcomes, DR progression, and HRQoL following SPKT in patients with advanced DR, a population rarely described in previous research.

## 5. Conclusions

In conclusion, SPKT improves metabolic control, stabilizes or improves DR, enhances visual acuity, and restores HRQoL to levels comparable with healthy, age-matched controls in most patients with T1D. The results of the current study indicate that DR progression may still occur years after achieving euglycemia, emphasizing the importance of long-term ophthalmic follow-up and monitoring after SPKT.

## Figures and Tables

**Table 1:** Clinical characteristics of participants after SPKT.

Characteristic	All (n = 18)	Stable DR (n = 11)	Progression of DR (n = 7)	*p*-Value
Sex, males, n (%)	12 (67)	6 (55)	6 (86)	0.171
Age at diagnosis of dm	8.5 (8.1) [1–34]	9.6 (9.1)	6.7 (6.6)	0.476
Duration of diabetes pre-SPKT	30.2 (8.6) [15–43]			
Age at SPKT, years (SD) [min-max]	38.7 (7.3) [29–53]	41.5 (7.9)	34.4 (3.4)	0.01 *
History of ketoacidosis, n (%)	10 (56)	7 (64)	3 (43)	0.387
Insulin treatment before SPKT				0.629
Multiple daily injections, n (%)	9 (50)	5 (46)	4 (57)	
Insulin pump, n (%)	9 (50)	6 (54)	3 (43)	
Duration of dialysis pre-SPKT, years (SD) [min-max]	1.5 (1.2) [0–4]	1.3 (0.8)	1.9 (1.6)	0.343
Dialysis type				0.557
Peritoneal, n (%)	8 (44)	4 (36)	4 (57)	
Hemodialysis, n (%)	10 (56)	7 (64)	6 (86)	
Duration of DR pre-SPKT, years (SD) [min-max]	16.0 (8.5) [1–31]	18.0 (9.3)	11.9 (5.2)	0.204
Severity of DR				0.688
NPDR, n (%)	2 (11)	1 (9)	1 (14)	
PDR, n (%)	16 (89)	10 (91)	6 (86)	
Previous treatments for DR				
Anti-VEGF-treatment, n (%)	9 (50)	5 (46)	4 (57)	0.629
Retinal photocoagulation, n (%)	16 (89)	10 (91)	6 (86)	0.732
Pars plana vitrectomy, n (%)	9 (50)	6 (55)	3 (43)	0.629
Previous medical history				
Medication for arterial hypertension, n (%)	18 (100)	11 (100)	7 (100)	-
Medication for dyslipidemia, n (%)	11 (61)	6 (55)	5 (71)	0.474
Cardiovascular disease, n (%)	5 (28)	5 (45)	-	0.351
Asthma, n (%)	1 (6)	1 (9)	-	0.412
Arthritis, n (%)	1 (6)	1 (9)	-	0.412
Diabetic polyneuropathy, n (%)	7 (39)	5 (45)	2 (29)	0.474
Charcot’s neuroarthropathy, n (%)	4 (22)	3 (27)	1 (14)	0.518
Anterior uveitis, n (%)	2 (11)	-	2 (29)	0.06
Secondary glaucoma, n (%)	2 (11)	1 (9)	1 (14)	0.732
SPKT associated adverse effects				
Chronic kidney transplant rejection, n (%)	3 (17)	1 (9)	2 (29)	0.28
Pancreas transplant rejection, n (%)	4 (22)	3 (27)	1 (14)	0.518
Post-transplant neutropenia, n (%)	1 (6)	-	1 (14)	0.197
Post-transplant lymphoma, n (%)	3 (17)	2 (18)	1 (14)	0.829
Immunosupressive therapy				0.434
MMF + TAC, n (%)	14 (78)	9 (82)	5 (71)	
MMF + TAC + PSL, n (%)	3 (16)	1 (9)	2 (29)	
AZA + TAC + PSL, n (%)	1 (6)	1 (9)	-	

SPKT, simultaneous pancreas–kidney transplantation; DR, diabetic retinopathy; NPDR, non-proliferative diabetic retinopathy; PDR, proliferative diabetic retinopathy; VEGF, vascular endothelial growth factor; MMF, mycophenolate mofetil; TAC, tacrolimus; PSL, prednisolone; AZA, azathioprine; SD, standard deviation. * *p* < 0.05 was considered statistically significant.

**Table 2:** Ophthalmological features and outcomes.

Ophthalmological Feature	All n = 18	Stable DR n = 11	Progression of DR n = 7	*p*-Value
Mean BCVA pre-SPKT, ETDRS letters (SD), [min–max], worse eye	64 (24) [0–90]	60.5 (27.9)	69.6 (15.8)	0.443
Mean BCVA post-SPKT, ETDRS letters (SD), [min–max], worse eye	69 (21) [0–85]	65.9 (25.9)	74.3 (11.7)	0.254
Rate of visual impairment pre-SPKT, n (%)	2 (11)	2 (18)	0	0.677
Rate of visual impairment post-SPKT, n (%)	1 (6)	1 (9)	0	0.412
Lense status after SPKT, worse eye				
No progression or previous IOL, n (%)	11 (61)	7 (64)	4 (57)	
Progression of cataract, n (%)	7 (39)	4 (36)	3 (43)	
CRT pre-SPKT, mean (SD), [min–max], worse eye	243 (36) [195–329]	234 (32)	257 (38)	0.217
CRT post-SPKT at the study visit, mean (SD), [min–max], worse eye	251 (46) [195–346]	232 (25)	307 (55)	0.29
Stability of DR post-SPKT, n (%)				
No treatment	11 (61)			
Macular laser	1 (6)			
Panretinal photocoagulation	2 (11)			
Anti-VEGF	1(6)			
Laser + anti-VEGF	3 (16)			
Pars plana vitrectomy	0 (0)			
Time to DR treatment after SPKT, months, median (IQR) [min–max]	9.9 (9.5) [2.7–68.3]			

BCVA, best corrected visual acuity; SPKT, simultaneous pancreas–kidney transplantation; ETDRS, Early Treatment Diabetic Retinopathy Study; SD, standard deviation; IOL, intraocular lens; CRT, central retinal thickness; DR, diabetic retinopathy; anti-VEGF, anti-vascular endothelial growth factor. *p* < 0.05 was considered statistically significant.

## Data Availability

The data presented in this study are available on request from the corresponding author.
